# A New Direction in Endometrial Cancer Therapy—PD-1 and PD-L1 Immune Checkpoint Inhibitors—Where Will It Take Us?

**DOI:** 10.3390/jcm14238366

**Published:** 2025-11-25

**Authors:** Natalia Mielnicka, Martyna Dutka, Krzysztof Kułak, Anna Kułak, Rafał Tarkowski

**Affiliations:** 1 Student’s Scientific Association, I Chair and Department and Clinic of Oncological Gynecology and Gynecology, Medical University of Lublin, 16 Staszica St., 20-081 Lublin, Poland; 2 I Chair and Department and Clinic of Oncological Gynecology and Gynecology, Medical University of Lublin, 16 Staszica St., 20-081 Lublin, Poland; 3 Department of Diagnostic and Microsurgery of Glaucoma, Medical University of Lublin, 1 Chmielna St., 20-079 Lublin, Poland

**Keywords:** endometrial cancer, immune checkpoint inhibitors, pembrolizumab, atezolizumab, dostarlimab

## Abstract

**Background**: Endometrial cancer (EC) remains a significant therapeutic challenge due to its rising incidence and a five-year survival rate of only 16% in cases of metastases or advanced disease when using classical therapy methods. For this reason, more effective methods of treatment are still sought, which currently focus on immunotherapy with the use of PD-1 and PD-L1 inhibitors (ICI), which is the subject of our article. **Methods**: Articles published in the databases: Pubmed, Web of Science, and ClinicalTrials.gov in the years 2019–2024 were analyzed. **Results**: ICIs can be used in monotherapy as well as in combination therapy, but it is the latter option that significantly prolongs the median PFS, especially the combination of ICI with PARP inhibitors. Among the available ICIs, pembrolizumab stands out with a large advantage in clinical trials, which is characterized by the lowest mortality resulting from therapy and a small number of grade 3 adverse events. Other inhibitors such as atezolizumab, dostarlimab, durvalumab, nivolumab and avelumab also demonstrate high clinical efficacy, as they prolong median PFS compared to the control group, but more studies are needed in much larger study groups to assess their safety and efficacy in different age groups. **Conclusions**: Future studies should focus on the efficacy of ICIs in younger groups of patients with EC, as well as on drugs from this group that are used less frequently in clinical trials than pembrolizumab, which would allow for a thorough comparison of the efficacy of drugs with each other and the selection of the drug individually to the patient’s needs.

## 1. Introduction

Endometrial cancer (EC) is the most common cancer of the female reproductive system, with a median diagnosis age of 61 years. Most cases occur in postmenopausal women, but 14% are diagnosed before menopause. Early symptoms include postmenopausal bleeding, intermenstrual bleeding, and prolonged heavy menstrual bleeding, while advanced stages may cause abdominal pain and urinary issues. EC has several histological subtypes, and its classification includes: endometrioid, serous, clear cell, mixed cell adenocarcinoma and other rare types. Incidence rates are rising, with 66,570 new cases in the U.S. in 2021. Risk factors include obesity, hypertension, insulin resistance, and genetic predisposition, while protective factors include higher parity and oral contraceptive use. According to statistics, the majority of EC diagnoses (67%) are localized. An important aspect is the 5-year survival rate, which for women diagnosed in the early stage of the disease is 95%, while for patients with metastatic or recurrent disease it is only 16% [1,2,3,4].

Depending on the stage of the disease, different methods of treatment are used. The FIGO scale, which was modified in 2023, is used to stage the disease. It allows for the assessment of the histological type, stage and lymphovascular space involvement (LVSI) [5,6]. The treatment of choice for localized EC is surgical resection of the tumor. The most common procedure is hysterectomy with bilateral salpingo-oophorectomy (BSO) [7,8]. Before the surgical procedure, imaging diagnostics are performed to determine the degree of invasion of the myometrium, cervix, and the presence of metastases in the lymph nodes. This allows for estimating the possibility of disease recurrence and determining the justification for surgical treatment [3]. Radiotherapy (RT) is currently used as an adjuvant treatment for intermediate- and high-risk EC. RT methods include external pelvic radiotherapy and vaginal brachytherapy. Both pelvic radiotherapy and vaginal brachytherapy are beneficial in reducing locoregional recurrence, most commonly in the vaginal vault. The advantage of brachytherapy is its lower gastrointestinal toxicity, which is why its use has become the norm in the treatment of patients with intermediate–high- or high-risk EC [8]. The global standard used in advanced EC is chemotherapy based on the combination of carboplatin and paclitaxel [9,10]. It replaced the previously used treatment regimen of paclitaxel-doxorubicin-cisplatin [11].

An important issue is to determine the status of DNA mismatch repair in the tumor by determining the molecular subtype of the tumor, which includes POLE ultramutation, MSI-H, p53 abnormal, and NSMP. Another classification also includes a dMMR subtype, instead of MSI-H [9,12]. It is estimated that 20–30% of patients with EC have mismatch repair deficiencies (dMMR) with concomitant high microsatellite instability (MSI-H) [12,13]. Tumors with dMMR show worse prognostic features compared to tumors with proficient mismatch repair (pMMR). Cancers with dMMR are larger in size and occupy a larger volume. As a result, they more often metastasize to lymph nodes [14]. In the NRG GOG210 study, a group of women with EC were analyzed. It was noted that patients diagnosed in more advanced stages of the disease, i.e., stage III, IV, had a more frequent dMMR tumor status [15]. Immune checkpoint inhibitors (ICI), which have achieved great clinical success in the treatment of many cancers, have recently also been used in EC therapy, constituting an innovative treatment method. The fact that PD-1 and PD-L1 are highly expressed in endometrial cancer (in approximately 40–80% of endometrial adenocarcinomas) speaks in favor of this therapy. By using PD-1 and PD-L1 inhibitors, it is possible to restore the cytotoxic activity of T lymphocytes, which stimulates the body’s anti-tumor response and gives a chance for a cure [1]. The growing number of EC cases each year is a serious public health problem, which is why it is necessary to modify the treatment and search for more effective therapies. We conducted a systematic review to outline the latest treatment options for endometrial cancer with PD-1 and PD-L1 inhibitors.

## 2. Material and Methods

**Figure 1:**
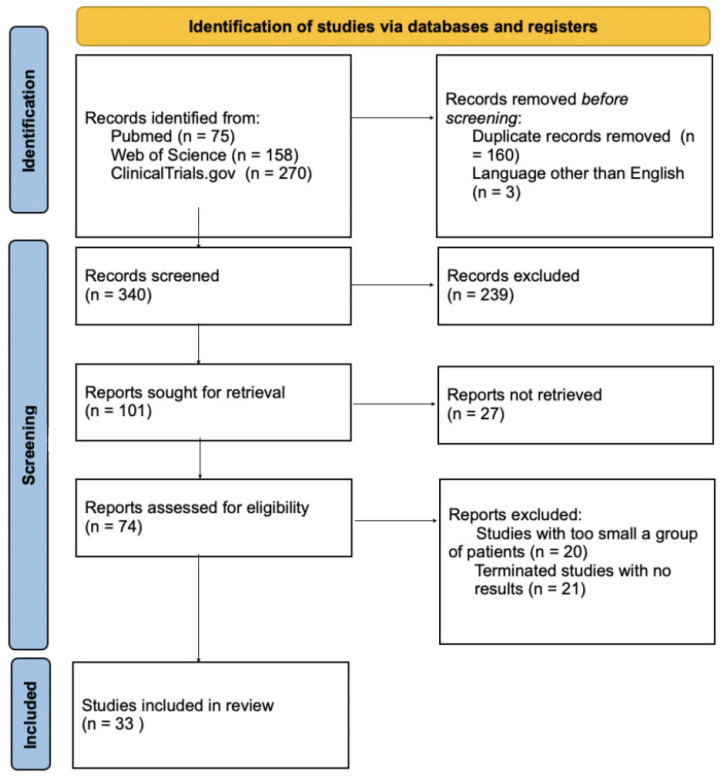
A flowchart presenting the process of study selection in accordance with PRISMA guidelines.

We conducted a detailed review of the literature using Pubmed, Web of Science, and ClinicalTrials.gov databases, considering articles written in English published between 2019 and 2024. We focused on recent studies that significantly contributed to clinical practice. Two researchers (N.M. and M.D.) conducted the database review independently and any doubts were mutually consulted and clarified. Inclusion criteria for this systematic review were as follows: randomized controlled trials, manuscripts, meta-analyses, systematic reviews, original articles and human studies. Exclusion criteria for this systematic review were as follows: case reports, articles not accessible as full-text, conference summary, and language other than English. The following search string was used: (“endometrial cancer” OR “endometrial tumor”) AND (“pembrolizumab” OR “nivolumab” OR “dostarlimab” OR “durvalumab” OR “atezolizumab” OR “avelumab” OR “immunotherapy” OR “PARP inhibitors” OR “multikinase inhibitors” OR “lenvatinib” OR “immune checkpoint inhibitors”). After applying inclusion and exclusion criteria, 33 articles were included for further analysis. This systematic review was conducted in accordance with the standards of preferred reporting items for systematic review and meta-analysis (PRISMA) [16]. All details of the entire study selection process are presented on the PRISMA flowchart (Figure 1).

## 3. Results

### 3.1. ICIs

EC immunotherapy uses a group of agents called ICIs (immune checkpoint inhibitors), which are a group of monoclonal antibodies directed against checkpoint proteins of the immune system. These include: pembrolizumab, atezolizumab, avelumab, nivolumab, dostarlimab and durvalumab [17].

The use of ICIs is caused by the fact that EC tumor cells are characterized by overexpression of PD-L1 and PD-L2 ligands in relation to PD-1 receptors, located on the surface of CD4+/CD8+ T lymphocytes, which infiltrate this tumor. This causes immunosuppression, resulting from the inability of T lymphocytes to function, and consequently further development of the tumor. ICIs block the reaction of PD-L1 and PD-L2 ligands with PD-1 receptors and restoring the cytotoxic activity of T lymphocytes [18].

ICIs are well suited for the treatment of advanced or recurrent EC, because early-stage cancer has lower PD-L1 expression, which results in lower efficacy of immunotherapy [19]. Moreover, their use was authorized after progression to platinum-based chemotherapy [20]. They can be used as a single-agent or combined with other medications.

### 3.2. Mechanism of Action of ICIs

**Figure 2:**
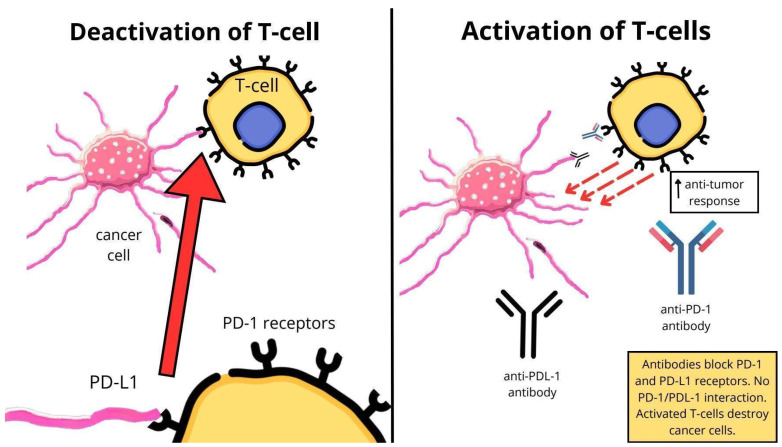
Mechanism of action anti-PD-1/PDL-1 antibodies own elaboration based on data from [23].

ICIs are a new type of cancer treatment. The mechanism of action of drugs from this group allows for more effective elimination of cancer cells [21]. PD-1 receptors are transmembrane proteins present on T lymphocytes and antigen-presenting cells (APCs). PD-L1 and PD-L2 molecules act as ligands for the PD-1 receptor and are localized on tumor cells and APCs. Most receptor–ligand interactions involve PD-1/PD-L1 because PD-L1 has a higher affinity for the receptor than PD-L2. Tumors have developed a mechanism to escape from immune control. Dysregulation of the MEK/ERK kinase pathway affects cell cycle progression and may carry the risk of malignant transformation. This leads to transcription of PD-L1 mRNA and expression of PD-L1 protein on the surface of tumor cells [22]. The PD-1/PDL-1 interaction has an immunomodulatory effect, leading to T cell deactivation and reduced antitumor activity. The secretion of proinflammatory cytokines IL-2, TNF-alpha, and INF-gamma is also increased. There is a loss of immune control over the tumor and acceleration of tumor aggression [22,23]. Immune checkpoints, including PD-1, are responsible for verification and selectivity towards appropriate cells. The association of the major histocompatibility complex (MHC) of the tumor cell with the T cell receptor (TCR) leads to the formation of the MHC-TCR complex. The MHC-TCR interaction enables the identification of cancer cells as abnormal and the activation of lymphocytes. Anti-PD-1/PD-L1 antibodies bind to the PD-1 and PD-L2 receptors and block their interactions. This allows the immune response to function properly. T lymphocytes are activated and the growth of cancer cells is inhibited [21]. In the rest of this article, ICIs used in the treatment of EC will be discussed in more detail, including their target molecules. Figure 2 shows the mechanism of action of PD-1/PDL-1 inhibitors. The figure was created in the graphic program Canva.

### 3.3. ICIs in Monotherapy

In the KEYNOTE-158 study conducted between 2016 and 2020, researchers evaluated the efficacy and safety of pembrolizumab for patients with MSI-H/dMMR EC whose previous treatment for advanced EC had failed, (most of them was radiotherapy). A total of 90 patients from 15 countries were divided into two cohorts: cohort D (MSI-H/dMMR-negative EC) and cohort K (MSI-H/dMMR advanced EC). Patients received 200 mg of pembrolizumab intravenously on day one of each three-week cycle for 35 cycles (approximately 2 years). Of the study group, 79 patients were included in the efficacy analysis, which showed that 48% of patients had an objective response to treatment (including 11 patients—14% had a complete response, and 27 patients—34% had a partial response). Additionally, 14 patients had stable disease, 13 of whom had a reduction in tumor size from baseline. As many as 88% of patients demonstrated an ongoing response of more than one year, followed by 73% of patients who maintained a response of more than two years, and 68% of patients who maintained a response of more than three years. The median progression-free survival was 13.1 months. The median overall survival was not reached. Adverse events occurred in 76% of patients, but only 12% of them were grade 3 or 4 adverse events. The most common were pruritus, fatigue, diarrhea, hypothyroidism, and hyperthyroidism. The study has shown high clinical efficacy with manageable toxicity in patients with advanced MSI-H/dMMR EC and with failed previous therapy [24]. With this study, the FDA granted approval for pembrolizumab monotherapy for advanced MSI-H/dMMR EC with disease progression [25].

The phase I GARNET study, conducted between 2017 and 2021, evaluated the use of dostarlimab in patients with advanced EC that had progressed on or after platinum therapy. Patients were divided into two cohorts: A1 (143 patients)—MSI-H/dMMR tumor and A2 (156 patients)—microsatellite stable tumors (MSS) or mismatch repair proficient tumors (pMMR). Patients received 500 mg dostarlimab every three weeks for four cycles, followed by 1000 mg every six weeks until progression. The objective response rate (ORR) was 45.5% in patients in cohort A1 and 15.4% in patients in cohort A2. The median duration of response (DOR) was not reached in cohort A1 and was 19.4 months in cohort A2. The median PFS was 6 months for cohort A1 and 2.7 months for cohort A2. An ongoing response was observed in both cohorts (patients in A1 had a 93.3% probability of maintaining a response beyond 12 months, compared to 60.3% probability in A2). The median OS was not reached in cohort A1, compared to 16.9 months in cohort A2. Adverse events included fatigue, diarrhea, nausea, anemia, hypothyroidism, arthralgia, increased ALT and AST. Sustained response rates were demonstrated in women with MSI-H/dMMR and MSS/pMMR EC and a manageable safety profile [26]. As a result of these studies, the EMA and FDA authorized dostarlimab for the treatment of patients with recurrent or advanced EC after prior platinum-based therapy [25].

Studies describing the use of ICIs in monotherapy are presented in Table 1.

### 3.4. ICIs and Chemotherapy

The randomized, double-blind, phase 3 RUBY study evaluated the safety and efficacy of dostarlimab in combination with carboplatin and paclitaxel for the treatment of advanced or recurrent EC from July 2019 to February 2021. The study included 494 patients divided into two groups: 245 received dostarlimab and chemotherapy, and 249 received placebo and chemotherapy (placebo group). It is worth adding that 53 people in the dostarlimab group had a dMMR/MSI-H tumor molecular type, while 65 in the placebo group had this type of tumor. Patients received 500 mg i.v. dostarlimab or placebo (depending on the group), in combination with carboplatin at a dose of 5 mg per milliliter per minute and paclitaxel at a dose of 175 mg per square meter of body-surface area intravenously every three weeks for the first six cycles, followed by 1000 mg dostarlimab or placebo i.v. every six weeks for three years or until disease progression, treatment discontinuation, or patient death. In the overall population, median PFS was 11.8 months for the dostarlimab group and 7.9 months for the placebo group. In the dMMR/MSI-H population, the estimated 24-month PFS was higher for the dostarlimab group (61.4%) than for the placebo group (15.7%). Median OS in the overall population was 44.6 months for dostarlimab versus 28.2 months for placebo (median follow-up of 37.2 months). Adverse events were similar in both the placebo and dostarlimab groups. The most common adverse events were nausea, alopecia, and fatigue. The most common adverse event in the dostarlimab group was maculopapular rash. This study confirmed the significant therapeutic benefit of combining dostarlimab with carboplatin and paclitaxel in patients with advanced and recurrent EC [27].

Another study, conducted between July 2019 and December 2022, evaluated the efficacy of standard chemotherapy in combination with pembrolizumab in patients with advanced or recurrent EC. It was a phase 3, international, randomized, double-blind study. A total of 816 patients were divided into two cohorts: 225 showed the dMMR molecular type, and 591 showed the pMMR type. In each cohort, patients were randomly assigned in a 1:1 ratio to two groups: patients in the first group received 200 mg of pembrolizumab i.v. in a 30 min infusion every three weeks in combination with chemotherapy followed by 400 mg of pembrolizumab i.v. in a 30 min infusion every 6 weeks, and patients in the second group received 200 mg of placebo i.v. in combination with chemotherapy every three weeks followed by 400 mg of placebo i.v. every six weeks. Chemotherapy consisted of paclitaxel at a dose of 175 mg per square meter of body-surface area administered intravenously in a 3 h infusion and carboplatin at a dose of 5 mg per milliliter per minute administered intravenously over 30–60 min. The median follow-up was 7.9 months in the pMMR cohort and 12 months in the dMMR cohort. The median PFS in the pMMR cohort was 13.1 months in the pembrolizumab group and 8.7 months in the placebo group. The 12-month PFS was 74% in the pembrolizumab group and 38% in the placebo group, demonstrating the great efficacy of this treatment option. The most common adverse events included fatigue, peripheral sensory neuropathy, anemia, nausea, constipation, and diarrhea. The results of this study show that the addition of pembrolizumab to chemotherapy and its continued use as maintenance therapy significantly prolongs the PFS of patients with dMMR and pMMR EC [9].

An interesting multicenter, randomized, double-blind AtTEnd study conducted between October 2018 and January 2022 analyzed the efficacy and safety of adding atezolizumab to chemotherapy in the treatment of advanced or recurrent EC. A total of 549 patients were randomly divided into two groups in a 2:1 ratio: atezolizumab group—360 patients and placebo group—189 patients. Patients received 1200 mg of atezolizumab (or placebo) i.v. in combination with carboplatin at an area under the curve of 5 or 6 and paclitaxel at a dose of 175 mg/m^2^ i.v. for 6–8 cycles, followed by 1200 mg of atezolizumab (or placebo) until disease progression or high toxicity of treatment. The dMMR molecular type was demonstrated by 81 patients in the atezolizumab group and 44 patients in the placebo group, and the pMMR molecular type was demonstrated by 269 patients in the atezolizumab group and 140 patients in the placebo group. In the overall population, median PFS was 10.1 months in the atezolizumab group compared with 8.9 months in the placebo group. Adverse events occurred with similar frequency in both the study and placebo groups, the most common of which were neutropenia, anemia, leukopenia, thrombocytopenia, febrile neutropenia, diarrhea, pneumonia and hypopituitarism. In patients with pMMR molecular type, atezolizumab treatment did not improve PFS or OS. The study demonstrated high efficacy of atezolizumab in combination with chemotherapy in patients with dMMR EC, while in pMMR tumors, this treatment did not produce the expected results [28] (Table 2).

### 3.5. ICIs and Multikinase Inhibitors

The need to find an effective EC treatment method prompted researchers to seek new combinations of therapy. A great example of this action is a study published in 2020, which analyzed the effectiveness and safety of the use of lenvatinib in combination with pembrolizumab in patients with advanced EC. Lenvatinib is a multikinase inhibitor that is directed against vascular endothelial growth factor receptors 1–3, platelet-derived growth factor receptor-α, fibroblast growth factor receptors 1–4, RET, and KIT. In this study, 108 patients received lenvatinib at a dose of 20 mg orally once a day and pembrolizumab at a dose of 200 mg intravenously once every three weeks in three weeks cycles. The median observation of patients was 18.7 months. The median PFS was 7.4 months, while the median OS 16.7 months. The most common side effects included: hypertension, diarrhea, fatigue, decreased appetite, hypothyroidism, and nausea. The study demonstrated the efficiency of the lenvatinib and pembrolizumab combination in patients with advanced EC who do not have the molecular subtype MSI-H or DMMR. Unfortunately, a significant limitation of this study was the lack of a control group that would allow for a comparison of median PFS between the two groups and to indicate whether the addition of lenvatinib to pembrolizumab actually increases median PFS [29].

Randomized study phase 3 309/KEYNOT-775 also analyzed the effectiveness of lenvatinib treatment in combination with pembrolizumab. A total of 827 patients were randomly assigned to one of the groups: 411 patients to a lenvatinib and pembrolizumab group, while 416 patients to a chemotherapy group. Patients from the first group received lenvatinib at a dose of 20 mg orally once a day and pembrolizumab at a dose of 200 mg i.v. once every three weeks. In turn, patients from the second group received chemotherapy, which consisted of doxorubicin 60 mg/m^2^ i.v. once every three weeks or paclitaxel 80 mg/m^2^ i.v. once a week. It is worth adding that 697 patients had a molecular type of pMMR tumor, while 130 people had a molecular type of dMMR tumor. The median observation was 18.7 months in the lenvatinib and pembrolizumab group and 12.2 months in the chemotherapy group. The percentage of patients with pMMR tumors with a confirmed objective response was higher in a group with lenvatinib plus pembrolizumab (32.4%) than in a group with chemotherapy (15.1%), with 5.8% and 2.6% of patients, respectively, achieving complete responses (CR). The median PFS for the group with lenvatinib and pembrolizumab was 7.3 months, while in a group with chemotherapy it was only 3.8 months. The most common side effects in the first group was hypertension, while in the group with chemotherapy it was anemia. This study confirms the high efficiency of the use of lenvatinib in combination with pembrolizumab in patients with advanced EC [10] (Table 3).

### 3.6. ICIs and PARP Inhibitors

PARP inhibitors have been used with great clinical success in ovarian cancer associated with mutations in the *BRCA1* and *BRCA2* genes. They capture the PARP protein, which is responsible for repairing single-strand DNA damage. PARP inhibition leads to the accumulation of double-strand DNA damage that cannot be repaired in cells with homologous recombination repair deficient (HRD), which represent cells with mutations in the *BRCA1* and *BRCA2* genes. The interest in PARP inhibitors in the context of endometrial cancer stems from the fact that HRD is associated with the *P53* mutation, which occurs in serous endometrial cancer, and in addition, HRD tumors are characterized by an increased immune response directed against cancer cells. An additional advantage of PARP inhibitors is the regulation of PD-L1 expression, which may additionally enhance the effect of PD-L1 inhibitors [30,31].

The phase III DUO-E study, a randomized, double-blind, global study, evaluated the efficacy and safety of durvalumab in combination with olaparib after prior therapy with durvalumab and carboplatin-paclitaxel. Patients were randomly assigned to 3 groups in a 1:1:1 ratio. In the control group, patients received carboplatin-based chemotherapy at a dose of 5 or 6 mg/mL/min once every three weeks for six cycles and paclitaxel at a dose of 175 mg/m^2^ once every three weeks for six cycles and durvalumab placebo i.v. and olaparib placebo orally. In the durvalumab cohort, patients received platinum-based chemotherapy and durvalumab at a dose of 1120 mg i.v. once every three weeks for six cycles, followed by durvalumab 1500 mg i.v. maintenance dose once every four weeks plus olaparib placebo p.o. In the durvalumab plus olaparib cohort, patients received platinum-based chemotherapy plus durvalumab 1120 mg i.v. once every three weeks for six cycles, followed by durvalumab 1500 mg i.v. maintenance dose once every four weeks plus olaparib 300 mg orally twice daily. The results of the study were promising, namely the PFS in the durvalumab group was 10.2 months compared with the control group PFS of 9.6 months. In addition, the durvalumab plus olaparib group PFS was 15.1 months compared with the control group PFS of 9.6 months. In all cohorts, the most common adverse events reported included nausea, anemia, alopecia, fatigue, and neutropenia. This study confirmed the high clinical benefit of combining PARP inhibitors, immunotherapy and chemotherapy [32].

### 3.7. The Efficiency of Therapy and the Type of ICIs Used

When comparing the efficacy of different ICIs used in therapy, several aspects should be considered. The most important of these are the number of available studies for each drug, the size of the patient groups included in the study, the incidence of grade 3 or worse adverse events, the most common adverse events, mortality in the study groups, and median PFS. Table 4 shows the number of studies available on Clinicaltrials.gov for EC treatment with the six ICIs, as of 5 January 2025.

#### 3.7.1. Pembrolizumab

Pembrolizumab is a humanized monoclonal antibody of the IgG subclass 4. The antibody binds to PD-1 receptors and blocks the PD-1/PD-L1 pathway, which reduces tumor growth [33]. In 2014, pembrolizumab was approved by the Food and Drug Administration (FDA) for the treatment of unresectable or metastatic melanoma that has progressed despite therapy with ipilimumab [33,34].

The KEYNOTE-158 study evaluated the response to pembrolizumab in multiple cancer types. Patients enrolled in the study had advanced non-colorectal cancer with MSI-H/dMMR status who had failed prior therapy. The study had only one arm and no control group. The study enrolled 233 participants. Cohort D, which included patients with advanced EC, was the largest, with 49 patients. This group also had the highest rate of tumor size reduction, with 33 of 47 patients (70%) experiencing a ≥30% reduction in tumor size. An overall response (OR) to treatment was achieved in eight patients in cohort D, which was a very favorable result compared to other cancers. PD-1 checkpoint inhibitor therapy proved to be the most beneficial for patients with advanced EC. ORR = 57.1, median PFS = 25.7 months were the highest for cohort D. However, median OS was undeterminable for this group, which resulted from the time limitation of the study, which was conducted from 1 February 2016 to 8 May 2018 (data cut-off time 6 December 2018). The undeterminable median OS should undoubtedly be considered a positive result of the study. Over half of participants, 151 out of 233 (64.8%), experienced an adverse reaction to their treatment. Grade 3–4 treatment-related adverse events (TRAEs) occurred in 34 of 233 participants (14.6%). Twenty-two people (9.4%) had to discontinue treatment due to adverse events associated with the drug. Symptoms such as diarrhea, pruritus, asthenia, and fatigue were the most common adverse events. Eighteen patients (7.7%) experienced serious adverse events, and one patient died due to pneumonia, which was a complication of treatment. According to available knowledge, the results of the phase II KEYNOTE-158 study confirm the validity of the use of pembrolizumab in the treatment of cancers with the MSI-H/dMMR status, especially in advanced EC. The favorable result of this study undoubtedly influenced the further development of research on the use of the drug in the treatment of EC in a broader context [12].

Due to the large number of available studies on the use of pembrolizumab in the treatment of EC, we used randomized phase III trials with a large number of participants for our analysis. We selected five studies according to the selection criteria: free full text and manuscripts written in English.

The only U.S. FDA-approved immunotherapy for pMMR endometrial cancer is the combination of pembrolizumab and lenvatinib [35]. In 2023, updated data on efficacy and safety [36] for the 2022 analysis of study 309/KEYNOTE-775. The phase III trial compared lenvatinib plus pembrolizumab with standard chemotherapy of doxorubicin or paclitaxel in patients with advanced EC who had disease progression and had received at least one prior dose of platinum-based therapy. The trial was randomized, with 827 patients randomly assigned to two treatment arms. Arm A1 included 411 patients treated with lenvatinib plus pembrolizumab, while arm A2 included 416 patients treated with chemotherapy of the treating physician’s choice. The study was not blinded. The forms and schedules of treatment were different. Arm A1 received 20 mg orally once daily and pembrolizumab 200 mg IV once every three weeks, whereas arm A2 received doxorubicin 60 mg/m^2^ IV once every three weeks or paclitaxel 80 mg/m^2^ IV. once weekly, following a three weeks on and one week off schedule. Patients were stratified by tumor repair status. In total, 697 patients had pMMR tumors, 130 patients had dMMR tumors. pMMR status was 84.2% in arm A1 and 84.4% in arm A2. Of note, the median PFS rate was significantly better in the pembrolizumab plus lenvatinib arm compared with the chemotherapy arm. Patients in arm A1 had a median PFS of 7.3 months; patients with pMMR tumors had a median PFS of 6.7 months. Regardless of tumor repair status, PFS was almost twice as long compared with patients in arm A2, who had a median PFS of 3.8 months. Adverse events were a significant concern for patients receiving both therapies. Safety analysis was performed in 794 patients. Any adverse event occurred in 405 of 406 (99.8%) patients in arm A1 and 386 of 388 (99.5%) in arm A2. Adverse events that occurred in more than 40% of patients in the pembrolizumab plus lenvatinib group included hypertension, hypothyroidism, diarrhea, nausea, and decreased appetite. The most common adverse events in the chemotherapy group were anemia, nausea, neutropenia, alopecia, fatigue, constipation, and diarrhea. Grade 3 or higher adverse events were observed in 366 of 406 (90.1%) patients in arm A1; 26 patients (6.4%) died from treatment-related adverse events. In arm A2, grade 3 or higher adverse events were observed in 286 of 388 (73.7%) patients; 20 (5.2%) patients had grade 5 adverse events [10].

Another multicenter study KEYNOTE-868 was published in 2023. Its aim was to determine whether combined chemotherapy with pembrolizumab would provide significant clinical benefit in patients with stage III or IVa with measurable disease and patients with stage IVb recurrent EC. The study included 816 patients, who were divided into two cohorts, depending on the result of the immunohistochemistry performed. In total, 225 people were included in the dMMR cohort, of whom 112 received pembrolizumab and 113 received placebo. The pMMR cohort originally included 591 people; however, 3 of them were not subjected to statistical analysis of treatment efficacy because their MMR result was inconclusive. A total of 293 participants from the pMMR cohort received pembrolizumab and 295 placebo. The study was to be analyzed over time until 84 deaths or progressions occurred in the dMMR cohort and 196 in the pMMR cohort. For the first six cycles, which lasted three weeks, patients were to receive 200 mg of pembrolizumab or placebo IV in a 30 min infusion in combination with 175 mg/m^2^ of paclitaxel IV in a 3 h infusion and 5 mg/mL/min of carboplatin IV in a 30–60 min infusion. After six cycles, the dose of pembrolizumab or placebo was changed to 400 mg IV in a 30 min infusion, the doses of combination chemotherapy were not changed, and the cycle duration was extended to six weeks. Patients could receive a maximum of 14 maintenance cycles of this treatment. For the pMMR cohort, the median PFS of the pembrolizumab-treated group was 13.1 months, while for the placebo group it was 8.7 months. The placebo group in the dMMR cohort achieved a median PFS of 7.6 months, while for the pembrolizumab-treated group the indicator was not reached, the range was given (30.6 months-NR). The positive effect of pembrolizumab treatment in the dMMR group is illustrated by the analysis of 12-month PFS = 74%, the placebo group was almost 2-fold lower, PFS12 = 38%. Any treatment-related adverse events occurred in almost all study participants. However, the pMMR cohort was slightly more favorable, in which TEAEs were experienced by 93.5% of the study participants (258 of 276 people) in the pembrolizumab group and 93.4% (256 of 274 people) in the placebo group. Compared to the dMMR cohort, where 98.2% (107 of 109) of pembrolizumab patients and 99.1% (105 of 106) of placebo patients had any adverse events. The most common adverse events in all groups included fatigue, anemia, peripheral sensory neuropathy, and gastrointestinal symptoms such as nausea, constipation, and diarrhea. More grade 3 or higher adverse events were reported in the pembrolizumab groups, 69 of 107 (63.3%) in the dMMR cohort and 152 of 276 (55.1%) in the pMMR cohort. The placebo groups had better results in this parameter, 47.2% (50 of 105) in the dMMR cohort and 45.3% (124 of 274). TEAEs resulted in the death of 3 of the 215 patients (1.4%) in the dMMR cohort (one in the pembrolizumab group, two in the placebo group), whereas in the pMMR cohort, 8 of 550 patients (1.5%) died (six in the pembrolizumab group, two in the placebo group). The combination of pembrolizumab with chemotherapy provided clinical benefit regardless of tumor phenotype. The benefit was greater in tumors with deficient mismatch repair (dMMR) [9].

KEYNOTE-B21 is a notable study published in 2024. The study included 1095 patients with newly diagnosed high-risk EC. Patients were eligible if their disease was at high risk of recurrence, which is defined as stage I/II disease with a p53/TP53 mutation and endometrioid histology or sarcoma and stage III/IV disease regardless of histology. Patients had to have had BSO and a good prognosis. No regional or distant metastases. In addition, they could not have received prior adjuvant therapy [37]. In contrast to the two previous studies, patients with recurrent disease were disqualified from the study [9,10]. KEYNOTE-B21 was a two-arm study. Patients were randomly assigned, with 545 assigned to pembrolizumab and 550 to placebo. In addition, patients were stratified by tumor MMR status. Of the 1095 patients, 281 (26%) had dMMR status and 814 (74%) had pMMR status [37]. This selection ratio based on tumor repair status is consistent with previously published epidemiological data. One study reported that pMMR repair status affects more than 70% of EC tumors [38]. The authors chose to investigate whether pembrolizumab plus chemotherapy and possibly additional radiotherapy would affect disease-free survival (DFS) in this group of patients. Six cycles of chemotherapy (5 or 6 mg/m^2^ of carboplatin IV and 175 mg/m^2^ of paclitaxel IV every 3 weeks) were planned. In case of severe hypersensitivity reactions to carboplatin and paclitaxel, cisplatin (75 mg/m^2^ i.v. every 3 weeks) or docetaxel (75 mg/m^2^ i.v. every 3 weeks) could be substituted; changes required discussion with the study sponsor. Pembrolizumab (or placebo) treatment was initiated at the same time at a regimen of 200 mg pembrolizumab (or placebo) every three weeks for six cycles. Then, at the discretion of the investigator, patients could receive external radiotherapy or four additional cycles of chemotherapy. They were treated with chemo-radiotherapy (50 mg/m^2^ i.v. cisplatin on days 1 and 29 after completion of six cycles of chemotherapy). In addition, after completion of six cycles of chemotherapy, patients were to receive 400 mg pembrolizumab (or placebo) every six weeks for six cycles. Any TRAEs occurred in all participants in both groups. Grade ≥ 3 AEs were reported in 386 of 543 (71%) participants in the pembrolizumab group and 348 of 549 (63%) in the placebo group. AEs related to the immune response to the infusion occurred in 228 of 543 (42%) patients in the pembrolizumab group and 133 of 549 (24%) patients in the placebo group. Treatment-emergent adverse events that occurred in both groups were similar. Alopecia and anemia affected more than half of the participants. Participants also complained of peripheral neuropathy, decreased white blood cell counts (particularly neutrophils), arthralgia, and gastrointestinal problems such as nausea, diarrhea, or constipation. During the study, 107 of 1095 (10%) participants died. However, the authors reported that none of the deaths were due to treatment-related side effects [37]. The KEYNOTE-868 and KEYNOTE-B21 studies were double-blind to reduce the risk of bias, which also increases the confidence in the results [9,37].

#### 3.7.2. Atezolizumab

Atezolizumab is a humanized engineered IgG1 monoclonal antibody directed against PD-L1, which is why it has a different mechanism of action than anti-PD-1 antibodies. It blocks the interaction of PD-1 and PD-L1, which restores T lymphocyte activity, and additionally blocks the interaction of PD-L1 and B7-1, which enhances the immune response [39]. In a study published in 2024, 549 patients were divided into two groups, of which one group (360 patients) received 1200 mg of atezolizumab i.v. in combination with chemotherapy on day 1 every 21 days for 6–8 cycles, followed by 1200 mg of atezolizumab on day 1 every 21 days until disease progression or unacceptable toxicity, and the other group (189 patients) received 1200 mg of placebo i.v. in combination with chemotherapy on day 1 every 21 days for 6–8 cycles, next 1200 mg of placebo on day 1 every 21 days. Grade 3 or worse adverse events occurred in 92 (26%) patients in atezolizumab group and 26 (14%) patients in placebo group. These included: neutropenia, anemia, leukopenia, thrombocytopenia, febrile neutropenia, fatigue, peripheral sensory neuropathy, pulmonary embolism and hypertension. Serious adverse events related to the treatment use occurred in 46 (13%) patients in the atezolizumab group and 6 (3%) patients in the placebo group. They included: diarrhea, pneumonia and hypopituitarism. In addition, grade 1–2 adverse events included cardiac failure, hypothyroidism, constipation, nausea, vomiting, pyrexia, urinary tract infection, decreased appetite, arthralgia, myalgia, paraesthesia, and alopecia. During the study, 148 (42%) patients in the atezolizumab group and 88 (47%) patients in the placebo group died. Median PFS in the atezolizumab group was 10.1 months, while in the placebo group it was 8.9 months. Despite the relatively high mortality, the use of atezolizumab prolonged PFS, which confirms its efficacy in this therapy [28].

#### 3.7.3. Dostarlimab

Dostarlimab is a humanized IgG4-k monoclonal antibody that binds to the PD-1 receptor, which blocks the receptor from binding to its ligands PD-L1 and PD-L2 [26]. In 2023, a study was published in which 494 patients were divided into two groups: 245 patients in the dostarlimab group (A1) and 249 patients in the placebo group (A2). Patients in group A1 received 500 mg of dostarlimab i.v. in combination with 5 mg/mL/min carboplatin and 175 mg/per square meter of body-surface area i.v. paclitaxel every three weeks for the first six cycles, followed by 1000 mg of dostarlimab i.v. every six weeks for up to three years, and patients in group A2 received 500 mg of placebo i.v. in combination with 5 mg/mL/min carboplatin and 175 mg/per square meter of body-surface area i.v. paclitaxel every three weeks for the first six cycles, followed by 1000 mg of placebo i.v. every six weeks for up to three years. During the study, 70.5% of patients in the dostarlimab group and 59.8% of patients in the placebo group experienced grade 3 or worse adverse events, which included: anemia, neutropenia, neutrophil count decreased, lymphocyte count decreased, white-cell count decreased, hypertension, and hypokalemia. In addition, serious adverse events occurred, which in the dostarlimab group were 37.8% of patients and in the placebo group were 27.6% of patients. These included: sepsis, pulmonary embolism, pyrexia, dyspnea, muscular weakness, anemia, asthenia, and urinary tract infection. Other adverse events included: fatigue, alopecia, nausea, peripheral neuropathy, arthralgia, constipation, diarrhea, myalgia, hypomagnesemia, decreased appetite, and rash. During the study, 65 (26.5%) patients in the dostarlimab group and 100 (40.2%) patients in the placebo group died. The median PFS in the dostarlimab group was 11.8 months, while in the placebo group it was 7.9 months. In this study, the significant difference in mortality between the two groups translated into a major clinical success, which is the endpoint (median PFS), which was higher in the dostarlimab group [27].

In another study published in 2023, a group of 299 patients was divided into two cohorts: cohort one (A1)—143 patients with dMMR/MSI-H EC and cohort two (A2)—156 patients with MMRp/MSS EC. Patients in both groups received 500 mg of dostarlimab every three weeks for four cycles, next 1000 mg of dostarlimab every six weeks. During the study, 17.6% of patients in cohort A1 and 20.5% of patients in cohort A2 experienced treatment-related adverse events grade 3 or worse. They included: ALT and AST increased, anemia, diarrhea, amylase increased, fatigue, hyperglycemia, lipase increased, and pneumonitis. Other adverse events that occurred included: hypothyroidism, nausea and asthenia. There were no deaths related to dostarlimab treatment during the study. Median PFS in A1 cohort was six months, and in A2 cohort was 2.7 months. Although the study showed greater efficacy in treating patients with dMMR/MSI-H EC, it lacked a control group to show whether it was worth exposing the patient to possible grade 3 adverse events resulting from the drug compared to the putative benefits of this therapy [26].

#### 3.7.4. Nivolumab

Nivolumab is a monoclonal antibody of the IgG4 subclass. It binds to the PD-1 receptor on T-lymphocytes and blocks the PD-1/PDL-1 pathway [33]. The multicenter NCI-MATCH (EAY131) screening study included patients with relapsed and refractory malignancies. A total of 4902 patients were examined. Molecular evaluation and tumors selection were performed using immunohistochemical methods. Nivolumab has been previously shown to be effective in dMMR colorectal cancer, so this group of patients was excluded from the study. The Z1D subprotocol is an open-label, single-arm, phase II clinical trial that ultimately enrolled 42 patients with dMMR tumors. The largest group consisted of patients with endometrioid adenocarcinoma of the endometrium (n = 13). Patients received nivolumab at a dose of 3 mg/kg i.v. every two weeks in 28-day cycles, with a change after the completion of four cycles. The dose was increased to 480 mg of nivolumab i.v. administered once every four weeks. Disease assessment was conducted every two cycles. The objective response rate (ORR) was 36% (15 of 42 patients), and the overall response (CR) was 7% (3 of 42 patients). The two patients with complete response had endometrioid adenocarcinoma. The median PFS was 6.3 months, and the median OS was 17.3 months. Twenty-two patients died during the study period, none due to treatment. The most common adverse events were fatigue, anemia, hypoalbuminemia and rash. The study demonstrated the potential efficacy of nivolumab in treating a range of dMMR tumors [40].

In 2022, the results of the phase II study were published. The efficacy of nivolumab in monotherapy and in combination with an anti-angiogenic drug was assessed. The study group consisted of women with recurrent, metastatic advanced endometrial cancer (aEC). Patients were randomly assigned in a 2:1 ratio; Arm A had 36 patients, and Arm B had 18 patients. Patients who had previously been treated with ICIs or whose tumor was carcinosarcoma were not eligible for the main arms of the study. Therefore, arm C was created for them, which initially included 23 people. The number of patients qualified for the study who underwent treatment was 77 people. The therapy cycle lasted 28 days. Patients in arms A and C received combination therapy, 40 mg of cabozatinib p.o. daily (the dose could be reduced to 20 mg in case of significant toxicity) and 240 mg of nivolumab i.v. on days 1 and 15 of the cycle. Patients in arm B received only nivolumab in the same doses and treatment schedule. After four cycles of therapy in all arms of the study, the dosage of nivolumab was to be changed to 480 mg every 28 days. In addition, patients from arm B whose condition had progressed could be transferred to arm C during the study. During the study, seven patients were added to arm C. The median PFS for arm A was 5.3 months, while for arm B it was 1.9 months. The median OS for arm A was 13.0 months, while for arm B it was 7.9 months, however this value is much less reliable due to the outflow of some patients to arm C. The ORR index was also more favorable in arm A = 25%, B = 11%. Interestingly, in arm C, the cohort that had previously been treated with immunotherapy, initially consisting of 13 patients, eventually 20, achieved an identically high ORR index as patients from arm A = 25%, who had no history of ICIs therapy. In terms of the occurrence of adverse effects of treatment, the combination therapy in arms A and C was much less favorable compared to monotherapy. Any adverse events were reported in 32 of 36 (89%) and 29 of 30 patients (97%), respectively. In both cohorts, more than half of the events were grade 3 or higher, 22 of 36 (61%) in arm A and 19 of 30 (63%) in arm C. The most common AEs in arm A patients were diarrhea, increased liver enzymes (ALT and AST), fatigue, hypertension, nausea, anorexia, weight loss, decreased platelet count, stomatitis, and hypothyroidism. Similar problems occurred with similar frequency in arm C patients. Two patients died from adverse events related to combination therapy. One assigned to arm A died of sepsis and one assigned to arm C died of tumor lysis syndrome. In the nivolumab monotherapy cohort, AEs were experienced by 12 of 18 patients (67%). Most of the adverse events were mild. Fatigue and nausea being the most common. Only one patient (6%) experienced a decrease in lymphocyte count classified as grade 3 or higher [41].

#### 3.7.5. Durvalumab

Two phase 2 studies were conducted to characterize the effects of durvalumab in combination therapy for recurrent, chronic advanced endometrial cancer (aEC) [42,43]. In the first study, durvalumab was administered in combination with tremelimumab (a CTLA-4 checkpoint inhibitor) [42] and in the second study, it was administered in combination with olaparib (a PARP inhibitor) [43]. Will the use of two checkpoint inhibitors with different mechanisms of action provide greater clinical benefit than monotherapy with one? This was the question posed by the investigators. The study was conducted at a single center only. Patients were randomized. Cycles lasted 28 days. Group 1 received 1500 mg of durvalumab every four weeks, whereas group 2 received 1500 mg of durvalumab and 75 mg of tremelimumab every four weeks. In group 2, only durvalumab was administered at the same frequency after four cycles. Patients were stratified by MMR status; 5 of 38 (13.2%) in group 1 and 4 of 39 (10.3%) had dMMR tumor status. Patients in both groups had a very poor response to treatment. In the monotherapy group, 4 of 37 (10.8%) participants had an objective response to treatment, and in the combination therapy group, 2 of 38 (5.3%) participants had CR. CR was noted only in those with dMMR tumor status, 2 in group 1 and 2 in group 2. The results of the study are unfavorable for both groups. The authors suggest that they may be related to the occurrence of resistance to immunotherapy treatment [42]. The DOMEC study was conducted in seven study centers. The study group consisted of 50 patients diagnosed with recurrent, metastatic, persistent EC. Patients had received at least one prior platinum-based chemotherapy regimen and could not have received prior PD-L1 or PARP inhibitors. Participants were required to receive durvalumab 1500 mg IV every four weeks and olaparib 300 mg orally twice daily until disease progression or unacceptable toxicity. The primary endpoint was defined as six months of progression-free survival. Treatment was considered effective if the PFS6 rate was 50% or greater. In the current study, PFS6 was reported in 17 of 50 (34%) patients. This is lower than was expected. The median PFS was 3.4 months. Of particular interest was the observation that among the 50 participants in the study, 10 were diagnosed with dMMR disease status. For this group, the median PFS was significantly higher at 5.5 months. In total, 44 of 50 (88%) experienced any side effects, but most were mild. More than 20% of study participants complained of nausea, fatigue, anemia, diarrhea, and anorexia. A total of 8 of the 50 (16%) patients had grade 3 adverse events, the most common of which was anemia. There were no treatment-related deaths [43].

The DUO-E trial, previously referenced, investigated the safety of conventional chemotherapy (carboplatin plus paclitaxel) in combination with durvalumab plus olaparib. Patients were assigned to three cohorts. The majority of study participants experienced adverse events (AEs). Fatigue, anemia, nausea, and alopecia were reported by more than 50% of respondents. The highest incidence of grade 3 or higher AEs was observed in the chemotherapy plus durvalumab plus olaparib group, with 160 of 238 (67%) participants experiencing these events. The study was large in size, and the mortality rate due to AEs due to TRAEs was 3.4% in the control group, 1.7% in the durvalumab group, and 2.1% in the durvalumab plus olaparib group [32].

#### 3.7.6. Avelumab

Avelumab is a monoclonal antibody belonging to the PD-L1 inhibitors. A phase II trial testing avelumab in the treatment of EC was published in 2019. Patients were divided into two cohorts. The determining factor was the MMR status of the tumor, confirmed by immunohistochemistry. Cohort I included 17 patients with dMMR tumors, and cohort II included 16 patients with pMMR tumors. Additionally, cohort I was to include patients with a POLE exonuclease domain mutation. Next-generation sequencing was performed and determined that no patient in either cohort had a POLE mutation. The initial assumptions had to be changed. Patients with relapsed EC who had measurable disease and had received at least one cycle of chemotherapy were eligible for the study. Participants were given 10 mg/kg intravenously avelumab every two weeks until progression or unacceptable toxicity. One of the primary endpoints was objective response rate. ORR was significantly better in cohort I (dMMR) in 4 of 15 (26.7%) patients, of whom one patient achieved a complete response. In cohort II (pMMR/non-POLE), only one patient had a partial response. Six-month PFS was also better in cohort I, at 40% (7 of 15 participants), with a median PFS of 4.4 months. In cohort II, it was 6.25% (1 of 16 participants), with a median PFS of 1.9 months. Any adverse events occurred in 22 of 31 patients (71%). Adverse events that occurred in more than one patient were fatigue, nausea, hypothyroidism, decreased neutrophil count, anemia, and diarrhea. Most AEs were not serious, with only 6 of 31 (19.4% of participants) experiencing grade 3 AEs. Patients with a dMMR mutation had significantly greater clinical benefits [44].

Monotherapy with checkpoint inhibitors shows low efficacy in pMMR tumors. An open-label, single-arm, controlled phase II study addresses this issue. The authors wanted to determine whether avelumab immunotherapy plus talazopazrib would provide significant clinical benefit in patients with relapsed pMMR EC. The repair status of the tumors was confirmed immunohistochemically. The study included 35 women. Patients received 1 mg of talazoparib orally daily and 10 mg/kg of avelumab every two weeks until disease progression or unacceptable toxicity. ORR was noted in four patients (11.4%), but none of them had a complete response to treatment. The median PFS was 3.6 months. One of the primary endpoints was 6-month PFS, which was 22.9%. The number of patients who experienced any TEAEs was not precisely specified, only a broad range of 10% or more was given. The most common treatment-related AEs were hematological disorders: anemia, thrombocytopenia, and neutropenia. Of the 35 patients, 9 (25.7%) experienced serious adverse events. The authors reported that no patient discontinued treatment due to AEs, but six patients (17%) had their medication doses reduced due to toxic effects. No patients died from this cause [35].

Supplement S1 presents studies on the above-described monoclonal antibodies that were used in the treatment of EC.

### 3.8. Resistance to Immunotherapy

Depending on the monoclonal antibody and combination therapy with lenvatinib, complete response (CR) in the treatment of advanced, recurrent endometrial cancer in clinical trials ranged from 5% to 17% [10,12,43]. According to previous knowledge, complete response to PD-1/PD-L1 pathway inhibitors is more frequently observed in patients with mismatch repair deficient (dMMR) tumors. However, the occurrence of resistance and unsatisfactory treatment outcomes even in this group of patients is becoming a serious problem [42]. When the body stops responding to immunotherapy, we are dealing with a secondary type of resistance [45]. The results of the phase II study are all the more interesting. Patients who had stopped responding to nivolumab monotherapy since progression were included in arm C. This group had been treated with ICI in the past and, as part of the study, with a combination of nivolumab and cabozantinib. After adding cabozantinib, patients with nivolumab resistance had an ORR of 25% with the combination [41]. In another study, patients with confirmed relapsed/metastatic dMMR EC were treated with 200 mg pembrolizumab IV (30 min infusion) every three weeks for two years or until progression. Four primary resistances (16.6%) and seven secondary resistances (29.2%) occurred in 24 participants [46]. The mechanisms of primary resistance are more complicated and are based on genetic mutations. In addition, ICIs can stimulate the expression of INF-γ, which through activation of the JAK-STAT pathway can lead to hyperprogression [45].

It is also worth mentioning the important financial toxicity of immunotherapy, meaning the negative impact of treatment costs on patients’ quality of life. Despite proven clinical effectiveness, the high cost of treatment significantly burdens patients and healthcare systems, prompting analyses to consider not only clinical outcomes but also the economic and ethical aspects of this therapy’s availability [45].

## 4. Discussion

Despite the constantly evolving treatment methods, endometrial cancer is still a challenge for doctors, due to the constantly growing number of cases and the low 5-year survival rate in the case of advanced stage or when there are metastases. Analysis of the literature on the use of immunotherapy in EC has led to many questions. Are PD-L1 and PD-1 inhibitors able to replace available and more well-known forms of treatment for this cancer? Which of the immune checkpoint inhibitors described in this review seems to be the most effective?

Chemotherapy together with radiotherapy and surgical treatment are the treatment of choice for EC, but when the disease is more advanced or there are metastases, these methods are unfortunately not effective. It is worth noting that the adding of ICIs to therapy has given patients a chance and hope for recovery, which is a huge advantage of these drugs. When reviewing current studies on them, a significant extension of the median PFS can be seen, including in the 2023 study by Eskander R.N. et al., which showed an increase in PFS with pembrolizumab to 13.1 months compared to the placebo group, where PFS was 8.7 months. Furthermore, this study showed that chemotherapy alone was not sufficient, as a much higher median PFS was achieved with the addition of ICI [9].

Most studies were conducted on groups of patients over 18 years of age but looking at the age structure of the study population, there is a significant dominance of people over 40 years of age. This may be a kind of limitation of these studies, as there is a lack of clear data on the effectiveness of immune checkpoint inhibitors in patients with EC aged 18–39, a younger population. This is important because increasingly younger women are being diagnosed with EC, and knowledge about the effectiveness of this treatment in this group of patients would perhaps allow this treatment to be introduced into therapy much faster. At this stage, it can only be speculated that the immune system in this age group may respond more strongly to ICI treatment, which may result in both greater treatment efficacy (translating into prolonged PFS) and, conversely, they may experience more intense or more complex adverse events following treatment. Studies on the effectiveness of ICIs in EC focusing on patients aged 18–39 would provide a clear answer.

Patient selection based on the molecular tumor MMR status is crucial in the context of immunotherapy with checkpoint inhibitors (ICIs). This has not resulted in significant clinical improvement in an unselected population [12]. Patients with dMMR tumors had significantly longer progression-free survival (PFS) compared with patients with pMMR tumor status, emphasizing the importance of patient selection based on tumor MMR status [26,44].

A controversial issue regarding the use of ICIs seems to be the numerous side effects that accompany this treatment; however, in our opinion, the clinical benefits resulting from their use significantly outweigh the possible side effects because there are drugs available that can reduce them to some extent, e.g., antiemetics. Moreover, the frequency of serious adverse events is not dominant when it comes to all side effects.

To clearly determine whether ICIs can replace current EC treatment methods, more studies are needed to analyze their use in monotherapy, because the current literature review leads us to reflect that patients benefit the most from combined therapies, with the most effective combination seeming to be the use of ICIs and PARP inhibitors, where the median PFS was extended by as much as five months [32].

Another important clinical aspect is the fact that when selecting therapy for a patient, a decision must be made as to which drug to choose. It is worth noting the significant advantage in the results of studies of pembrolizumab, which of all the ICIs described by us has the largest number of studies available, and recruitment is also being conducted for many future studies. Mortality among patients using pembrolizumab is not as high as, for example, atezolizumab, where in the study by Colombo N. et al. almost half of the patients using this drug died. The reason could be that 148 patients had stage IV disease, so the disease was already so advanced that treatment was ineffective or that the ICI dose for this group of patients was too high and should be reduced. Perhaps such an action would have reduced mortality in this study. The results of this study may therefore discourage physicians from using atezolizumab in therapy, so in the future it may be considered to conduct a clinical trial using this drug, but at a lower dose [28].

Choosing an ICI can be a challenge, as some studies had limitations. Oaknin A et al.’s 2023 study of dostarlimab did not have a control group to clearly determine whether this drug actually provides clinical benefit over placebo and whether it is worth exposing patients to the grade 3 adverse events that can occur with this therapy [26]. Similarly, the KEYNOTE-158 study, which examined the efficacy of pembrolizumab, did not have a control group and was a single-arm study, so it is difficult to compare the results of this study with other therapies without having a reference point as to whether it actually prolongs the median PFS [12]. The studies on avelumab also did not have a control group, and additionally the group of patients included in the study consisted of just over 30 people, so it is difficult to analyze these studies in terms of the actual effectiveness of this ICI, because a larger population of studied patients correlates with a greater likelihood of more frequent occurrence of adverse events, and then the analysis of these studies would have completely different results [35,44].

Considering the limitations of the studies presented, the conclusion is that pembrolizumab, on which the largest number of studies have been conducted, shows the greatest efficacy in treatment. In addition, its safety seems to be the highest among all the ICIs we have mentioned, which results from the fact that most of the studies on it had a control group (which is why the difference in median PFS was evidence of the efficacy of this drug), and additionally, the studies were conducted on very large groups of patients (the number was even over 1000 patients). However, in the long term, we cannot limit ourselves to the use of pembrolizumab, because the rest of the drugs we have mentioned also have great clinical potential (dostarlimab, atezolizumab, durvalumab, nivolumab). However, more studies on them are needed, with larger groups of patients and, importantly, with randomization and a control group, as well as at least double-blinding. This would allow for a reliable comparison of these drugs and their individual adjustment to each patient.

## 5. Conclusions

The literature review found that ICIs are highly effective and safe for the treatment of advanced or recurrent endometrial cancer. However, further research is needed to assess their efficacy in younger patients. Furthermore, more studies should focus on less commonly used drugs from this group, which may demonstrate even greater clinical success and give patients a chance for a cure, as current studies lead to the conclusion that pembrolizumab is the most effective and safe. Currently, there is little available research on the mechanisms of resistance to ICIs in endometrial cancer. Future research should draw attention to the problem of development of resistance to treatment with checkpoint inhibitors to avoid limited response to treatment. Furthermore, combination therapies appear to be promising, with immunotherapy plus chemotherapy proving the most effective, achieving the greatest improvement in PFS at 13.1 months, a finding supported by the fact that such treatment resulted in the fewest adverse events. However, this does not exclude the possibility that ICIs will be able to completely replace currently used EC therapies in the near future; more research is needed for this.

## Figures and Tables

**Table 1:** Use of ICIs in monotherapy.

Author of the Study	Year of Publication	Medicine and Dose	Number of Patients	Adverse Events	Median PFS
O’ Malley D.M. et al. [24]	2022	200 mg of pembrolizumab i.v. every 3 weeks for 35 cycles	79	pruritus, fatigue, diarrhea, hypothyroidism, hyperthyroidism	13.1 months
Oaknin A. et al. [26]	2023	500 mg of dostarlimab every 3 weeks for 4 cycles, next 1000 mg of dostarlimab every 6 weeks	299 (cohort A1 [dMMR/MSI-H] = 143, cohort A2 [MMRp/MSS] = 156)	fatigue, diarrhea, nausea, hypothyroidism, arthralgia, increased AST and ALT, anemia	A1 cohort = 6 months A2 cohort = 2.7 months

**Table 2:** Use of ICIs in combination with chemotherapy.

Author of the Study	Year of Publication	Medicine and Dose	Number of Patients	Adverse Events	Median PFS
Mirza M.R. et al. [27]	2023	500 mg of dostarlimab (or placebo) i.v. in combination with 5 mg/mL/min carboplatin and 175 mg/per square meter of body-surface area i.v. paclitaxel every 3 weeks for the first 6 cycles, next 1000 mg of dostarlimab (or placebo) i.v. every 6 weeks for up to 3 years	494 (245—dostarlimab group; 249—placebo group)	nausea, alopecia, fatigue, maculopapular rash	dostarlimab group = 11.8 months placebo group = 7.9 months
Eskander R.N. et al. [9]	2023	200 mg of pembrolizumab (or placebo) i.v. in a 30 min infusion every 3 weeks in combination with chemotherapy, next 400 mg of pembrolizumab (or placebo) i.v. in a 30 min infusion every 6 weeks	816 (225—EC dMMR, 591—EC pMMR)	fatigue, peripheral sensory neuropathy, anemia, nausea, constipation, diarrhea	pMMR cohort: -pembrolizumab group = 13.1 months -placebo group = 8.7 months 12-month PFS in dMMR cohort: -pembrolizumab group = 74% -placebo group = 38%
Colombo N. et al. [28]	2024	1200 mg of atezolizumab (or placebo) i.v. in combination with chemotherapy on day 1 every 21 days for 6–8 cycles, next 1200 mg of atezolizumab (or placebo) on day 1 every 21 days until disease progression or unacceptable toxicity	549 (360—atezolizumab group, 189—placebo group)	neutropenia, anemia, leukopenia, thrombocytopenia, febrile neutropenia, diarrhea, pneumonia, hypopituitarism	atezolizumab group = 10.1 months, placebo group = 8.9 months

**Table 3:** Use of ICIs in combination with multikinase inhibitors.

Author of the Study	Year of Publication	Medicine and Dose	Number of Patients	Adverse Events	Median PFS
Makker V. et al. [29]	2020	20 mg of lenvatinib orally once a day in combination with 200 mg of pembrolizumab i.v. once every 3 weeks in 3-week cycles	108	hypertension, diarrhea, fatigue, decreased appetite, hypothyroidism, nausea, stomatitis, decreased appetite, dysphonia, arthralgia	7.4 months
Makker V. et al. [10]	2023	A1: 20 mg of lenvatinib orally once a day in combination with 200 mg of pembrolizumab i.v. once every 3 weeks in 3-week cycles A2: doxorubicin 60 mg/m^2^ i.v. once every 3 weeks or paclitaxel 80 mg/m^2^ i.v. once a week	827 (416-chemotherapy group [A1], 411-lenvatinib and pembrolizumab group [A2])	hypertension, anemia, hypothyroidism, diarrhea, nausea, decreased appetite, vomiting, fatigue, arthralgia, proteinuria, constipation, headache	A1 group = 7.3 months A2 group = 3.8 months

**Table 4:** Number of studies testing the use of ICIs in ECs.

Monoclonal Antibody	Pembrolizumab	Atezolizumab	Dostarlimab	Nivolumab	Durvalumab	Avelumab
All available trials *	72	12	17	24	15	4
Trials with results	12	0	0	5	2	1

* Excludes discontinued, withdrawn, and unknown trials.

## Data Availability

No new data were created or analyzed in this study. Data sharing is not applicable to this article.

## Supplementary Materials

The following supporting information can be downloaded at: https://www.mdpi.com/article/10.3390/jcm14238366/s1, Supplement S1: Summary of key studies included in the review.

## Abbreviations

The following abbreviations are used in this manuscript:

PRISMA	Preferred Reporting Items For Systematic Reviews And Meta-Analyses
EC	Endometrial Cancer
PFS	Progression-free survival
OS	Overall Survival
ORR	Objective Response Rate
DFS	Disease-free survival
TRAE	Treatment-related adverse event
AE	Adverse event
i.v.	intravenous
