# Supplementary material for: A New Direction in Endometrial Cancer Therapy—PD-1 and PD-L1 Immune Checkpoint Inhibitors—Where Will It Take Us?
Source: J Clin Med. 2025 Nov 25;14(23):8366. doi: 10.3390/jcm14238366 (PMC12693102; doi:10.3390/jcm14238366)
Supplement: Supplementary file 1 [file jcm-14-08366-s001.zip › jcm-3931482-supplementary.pdf]

Supplement S1. Summary of key studies included in the review.

Author of the study	Year of publication	Medicine and dose	Numbers of patients	Frequency of adverse events (AEs)	Adverse events	Number of deaths during the study (including ICI use)	Median PFS
Marabelle A. et al.[12]	2020	200 mg pembrolizumab i.v. once every 3 weeks for 35 cycles or until documented disease progression	Total (n = 233)<br>- patients with advanced EC: 49	151 (64.8%) patients<br>34 (14.6%) patients experienced grade 3 to 5 TRAEs	fatigue, pruritus, diarrhea, asthenia, hypothyroidism, arthralgia, nausea, rash	113 (48,5%)<br>1 death related to AEs of treatment	all types of cancer: 4.1 months<br>endometrial cancer: 25.7 months
Makker V. et al.[10]	2023	A1: 20 mg of lenvatinib p.o. once daily and 200mg of pembrolizumab i.v. every 3 weeks<br>A2: 60mg/m² of doxorubicin i.v. every 3 weeks or 80mg/m² of paclitaxel i.v. every week [on a cycle of 3 weeks of drug and 1 week off	Total (n = 827)<br>A1: 411<br>A2: 417<br>- 697 with dMMR tumors<br>- 130 patients with pMMR tumors	A1:<br>405 of 406 (99,8%) patients<br>A2:<br>386 of 388 (99,5%) patients	A1:<br>hypertension, hypothyroidism, diarrhea, nausea, decreased appetite, vomiting, weight decrease, fatigue, arthralgia<br>A2:<br>anemia, nausea, neutropenia, alopecia, fatigue, constipation, diarrhea	A1: 26 of 405 (6,4%) of patients treated with the combination of pembrolizumab and lenvatinib died as a result of treatment<br>A2: 20 of 388 (5,2%) of patients patients who received chemotherapy died as a result of treatment	A1 group: 7. 3 months<br>A2 group: 3. 8 months
Eskander R.N. et al.[9]	2023	200 mg pembrolizumab (or placebo) i.v. in a 30-minute infusion in combination with 175 mg/m2 paclitaxel per body surface area i.v. in a 3-hour infusion and 5 mg/ml/min carboplatin i.v. in a 30-60 minute infusion every 3 weeks, followed after 6 cycles by 400 mg pembrolizumab (or placebo) i.v. in a 30-minute infusion in combination with chemotherapy every 6 weeks	Total (n = 816)<br>pMMR tumors (n = 591)<br>- pembrolizumab group: 295 patients<br>- placebo group: 296 patients<br>dMMR tumors (n = 225)<br>- pembrolizumab group: 112 patients<br>- placebo group: 113 patients	pMMR cohort: pembrolizumab group - 258 (93.5%)<br>placebo group - 256 (93.4%)<br>dMMR cohort: pembrolizumab group - 107 (98.2%)<br>placebo group - 105 (99.1%)	fatigue, peripheral sensory neuropathy, anemia, nausea, constipation, diarrhea	2 deaths as a result of AEs:<br>3 of 215 patients (1.4%) in the dMMR cohort,<br>(1 in the pembrolizumab group, 2 in the placebo group)<br>8 of 550 patients (1.5%) in the pMMR cohort, 6 in the pembrolizumab group and 2 in the placebo group	pMMR cohort:<br>- pembrolizumab group: 13.1 months<br>- placebo group: 8.7 months<br>dMMR cohort:<br>- pembrolizumab group: NR<br>- placebo group: 7.6 months

Van Gorp T. et al.[37]	2024	6 cycles of chemotherapy (5 or 6 mg/m2 carboplatin and 175 mg/m2 paclitaxel i.v. every 3 weeks)*. followed by external beam radiotherapy or radiochemotherapy with 50 mg/m2 cisplatin administered on days 1 and 29 after completion of 6 cycles of chemotherapy**. A1: pembrolizumab 200 mg i.v every 3 weeks for 6 cycles, then 400 mg every 6 weeks for 6 cycles A2: placebo every 3 weeks for 6 cycles, followed by a further 6 cycles every 6 weeks	Total (n = 1,095) - pembrolizumab group: 545 patients - placebo group: 550 patients	pembrolizumab group: 543 of 543 (100%) patients experienced any AEs, 386 of 543 patients (71%) experienced grade 3 or higher AEs placebo group: 547 of 547 (100%) patients experienced any side effects, 348 of 547 patients (63%) experienced grade 3 or higher adverse events	alopecia, anemia, nausea, diarrhoea, decreased WBC count, decreased neutrophil count, constipation, peripheral neuropathy, arthralgia	107 (10%) 0 deaths as a result of AEs	DFS of pembrolizumab group: 119 (22%) DFS in placebo group: 122 (22%)
Colombo N. et al.[28]	2024	1200 mg of atezolizumab (or placebo) i.v. in combination with chemotherapy on day 1 every 21 days for 6-8 cycles, next 1200 mg of atezolizumab (or placebo) on day 1 every 21 days until disease progression or unacceptable toxicity	Total (n = 549) - atezolizumab group: 360 patients - placebo group: 189 patients	grade 3 or worse (consequently study treatment): 92 patients (26%) in atezolizumab group, 26 patients (14%) in placebo group; serious adverse events related to the treatment use: 46 patients (13%) in the atezolizumab group and 6 patients (3%) in the placebo group	neutropenia, anemia, leukopenia, thrombocytopenia, febrile neutropenia, diarrhoea, pneumonia, hypopituitarism, fatigue, peripheral sensory neuropathy, pulmonary embolism, hypertension, cardiac failure, hypothyroidism, constipation, nausea, vomiting, pyrexia, urinary tract infection, decreased appetite, arthralgia, myalgia, paraesthesia, alopecia	148 patients (42%) died in the atezolizumab group; 88 patients (47%) died in the placebo group	atezolizumab group: 10.1 months, placebo group: 8.9 months

Mirza M. R. et al.[27]	2023	500 mg of dostarlimab (or placebo) i.v. in combination with 5 mg/ml/min carboplatin and 175 mg/per square meter of body-surface area i.v. paclitaxel every 3 weeks for the first 6 cycles, next 1000 mg of dostarlimab (or placebo) i.v. every 6 weeks for up to 3 years	Total (n = 494)<br>- dostarlimab group: 245 patients<br>- placebo group: 249 patients	serious adverse events: 37,8% in the dostarlimab group and 27,6% in the placebo group; grade 3 or worse: 70,5% in the dostarlimab group and 59,8% in the placebo group	nausea, alopecia, fatigue, maculopapular rash, anemia, neutropenia, neutrophil count decreased, lymphocyte count decreased, white-cell count decreased, hypertension, hypokalemia, sepsis, pulmonary embolism, pyrexia, dyspnea, muscular weakness, asthenia, urinary tract infection, peripheral neuropathy, arthralgia, constipation, diarrhea, myalgia, hypomagnesemia, decreased appetite	65 patients (26,5%) died in the dostarlimab group and 100 patients (40,2%) died in the placebo group	dostarlimab group: 11.8 months<br>placebo group: 7.9 months
Oaknin A. et al.[26]	2023	500 mg of dostarlimab every 3 weeks for 4 cycles, next 1000 mg of dostarlimab every 6 weeks	Total (n = 299)<br>- cohort A1 [dMMR/MSI-H] : 143 patients<br>- cohort A2 [MMRp/MSS]: 156 patients	TRAEs grade 3 or worse: 17,6% patients in cohort A1 and 20,5% patients in cohort A2	fatigue, diarrhea, nausea, hypothyroidism, arthralgia, increased AST and ALT, anemia, amylase increased, hyperglycemia, lipase increased, pneumonitis, asthenia	no deaths related to dostarlimab treatment	A1 cohort: 6.0 months<br>A2 cohort: 2.7 months
Post C.C.B. et al.[42]	2022	1500 mg of durvalumab i.v. every 4 weeks and 300 mg of olaparib p.o. twice a day,<br>until unacceptable toxicity or disease progression	Total (n = 50)	44 of 50 (88%) patients, grade 3 TREAs occurred in 8 of 50 (16%) patients	fatigue, nausea, anemia, diarrhea, anorexia, vomiting, dysgeusia, renal complications, flu-like symptoms	0 deaths as a result of AEs	3.4 months<br>PFS 6: 34%

Rubinstein M.M. et al.[41]	2023	Arm 1: 1500 mg durvalumab i.v. every 4 weeks,<br>Arm 2: 1500 mg durvalumab and 75 mg tremelimumab i.v. every 4 weeks for a maximum of 4 cycles, then 1500 mg durvalumab every 4 weeks	Total (n = 77)<br>Arm 1: 38 patients<br>Arm 2: 39 patients	no specific percentage data	hyperglycemia, anemia, hypoalbuminemia, hypomagnesemia, decrease in the number of WBCs, especially lymphocytes, increasing AST level, fatigue	no data	Arm 1: 7.4 weeks<br>Arm 2: 7.9 weeks
Westin S.N. et al.[32]	2024	Control group: 5 or 6 mg/ml/min carboplatin and 175 mg/m2 paclitaxel once every 3 weeks for 6 cycles and olaparib and durvalumab placebo.<br>Durvalumab group: chemotherapy + 1120 mg durvalumab i.v. once every 3 weeks for 6 cycles, followed by 1500 mg durvalumab i.v. (maintenance dose) once every 4 weeks and olaparib placebo<br>Durvalumab and olaparib group: chemotherapy + 1120mg durvalumab i.v. once every 3 weeks for 6 cycles, followed by durvalumab at a maintenance dose of 1500mg i.v. once every 4 weeks and 300 mg olaparib p.o. twice daily	Total (n = 718; 710 received treatment)<br>Control arm: 241 patients (236 received treatment)<br>Durvalumab arm: 238 patients (235 received treatment)<br>Durvalumab + olaparib arm: 239 patients (238 received treatment)	Any TRAEs patients:<br>Control arm 236 of 236 (100%)<br>Durvalumab arm: 232 of 235 (98,7%)<br>Durvalumab + olaparib arm: 237 of 238 (99,6%)<br>3rd grade AEs or higher:<br>Control arm: 133 of 236 (56,4%)<br>Durvalumab arm: 129 of 235 (54,9%)<br>Durvalumab + olaparib arm: 160 of 238 (67,2%)	anemia, nausea, fatigue or asthenia, alopecia, neutropenia, constipation, thrombocytopenia, diarrhea, vomiting, neuropathy peripheral, peripheral neuropathy, arthralgia, decreased appetite, leukopenia, urinary tract infection	AEs leading to death:<br>Control arm: 3. 4%<br>Durvalumab: 1.7%<br>Durvalumab + olaparib: 2. 1%	Control group: 12.6 months<br>Durvalumab group: 15.4 months<br>Durvalumab + olaparib group: 15.4 months

Azad N.S. et al. [40]	2019	ZD1 arm:<br>3 mg/kg of nivolumab i.v. every 2 weeks,<br>after 4 cycles 480 mg i.v. every 4 weeks	Total (n = 42)<br>- patients with endometrioid adenocarcinoma of endometrium: 13	no specific percentage data	fatigue, anemia, rash, hipoalbuminemia	22 (52,4%)<br>0 deaths as a result of AEs	6.3 months<br>PFS 6: 51,3%<br>PFS 12: 46,2%<br>PFS 18: 31,4%
Lheureux S. et al.[41]	2022	Arm A and C:<br>40 mg cabozantinib p.o. daily and 240 mg nivolumab i.v. on days 1 and 15 of the cycle.<br>B-Arm: 240 mg nivolumab i.v. on days 1 and 15 of the cycle,<br>after 4 cycles of therapy in all arms 480 mg nivolumab every 28 days.	Total (n = 77)<br>A-Arm: 36 patients<br>B-Arm: 18 patients<br>C-Arm: 30 patients	A-arm:<br>32 of 36 (89%)<br>B-arm:<br>12 of 18 (67%)<br>C-arm:<br>29 of 30 (97%)	Arm A and C:<br>diarrhoea, increased of ALT and AST, fatigue, hypertension, anorexia, nausea, weight loss, oral mucositis, decreased platelet count hypothyroidism<br>B-arm: fatigue, nausea	2 deaths as a result of AEs<br>A-Arm: 1<br>C-Arm: 1	A-Arm: 5.3 months<br>B-Arm: 1.9 months
Konstantinopoulos A. et al. [44]	2019	10 mg/kg of avelumab i.v. every 2 weeks until progression or unacceptable toxicity	Total (n = 31)<br>cohort I (dMMR): 15 patients<br>cohort II (pMMR): 16 patients	22 (71%),<br>TRAEs 3 grade had 6 of 31 (19.4%) patients	fatigue, nausea, hypothyroidism, neutrophil count decreased, anemia, diarrhea	0 patients has died from AEs	p-MMR/non-POLE: 1.9 months<br>d-MMR: 4.4 months
Konstantinopoulos A. et al. [35]	2022	1 mg of talazoparib p.o./ everyday and 10 mg/kg i.v. avelumab every 2 weeks until disease progression or unacceptable toxic effects occurred	Total (n = 35)	9 of 35 participants (25.7%) experienced severe TRAEs	anemia, thrombocytopenia, neutropenia	0 patients has died from AEs	3.6 months<br>PFS 6: 8 of 35 (22,9%)
